# CFTR Regulates Early Pathogenesis of Chronic Obstructive Lung Disease in βENaC-Overexpressing Mice

**DOI:** 10.1371/journal.pone.0044059

**Published:** 2012-08-24

**Authors:** Bjarki Johannesson, Stephanie Hirtz, Jolanthe Schatterny, Carsten Schultz, Marcus A. Mall

**Affiliations:** 1 Department of Translational Pulmonology, Translational Lung Research Center Heidelberg (TLRC), Member of the German Center for Lung Research, University of Heidelberg, Heidelberg, Germany; 2 Division of Pediatric Pulmonology and Allergy and Cystic Fibrosis Center, Department of Pediatrics III, University of Heidelberg, Heidelberg, Germany; 3 Cell Biology and Biophysics Unit, European Molecular Biology Laboratory, Heidelberg, Germany; 4 Molecular Medicine Partnership Unit, University of Heidelberg and European Molecular Biology Laboratory, Heidelberg, Germany; Johns Hopkins School of Medicine, United States of America

## Abstract

**Background:**

Factors determining the onset and severity of chronic obstructive pulmonary disease remain poorly understood. Previous studies demonstrated that airway surface dehydration in βENaC-overexpressing (βENaC-Tg) mice on a mixed genetic background caused either neonatal mortality or chronic obstructive lung disease suggesting that the onset of lung disease was modulated by the genetic background.

**Methods:**

To test this hypothesis, we backcrossed βENaC-Tg mice onto two inbred strains (C57BL/6 and BALB/c) and studied effects of the genetic background on neonatal mortality, airway ion transport and airway morphology. Further, we crossed βENaC-Tg mice with CFTR-deficient mice to validate the role of CFTR in early lung disease.

**Results:**

We demonstrate that the C57BL/6 background conferred increased CFTR-mediated Cl^−^ secretion, which was associated with decreased mucus plugging and mortality in neonatal βENaC-Tg C57BL/6 compared to βENaC-Tg BALB/c mice. Conversely, genetic deletion of CFTR increased early mucus obstruction and mortality in βENaC-Tg mice.

**Conclusions:**

We conclude that a decrease or absence of CFTR function in airway epithelia aggravates the severity of early airway mucus obstruction and related mortality in βENaC-Tg mice. These results suggest that genetic or environmental factors that reduce CFTR activity may contribute to the onset and severity of chronic obstructive pulmonary disease and that CFTR may serve as a novel therapeutic target.

## Introduction

Chronic obstructive pulmonary disease (COPD) characterized by airflow obstruction due to chronic bronchitis with airways mucus plugging and/or emphysema belongs to the most common chronic diseases and has evolved as a leading cause of death worldwide [1]. Although it is well established that most COPD is caused by cigarette smoke and exposure to other environmental pollutants, the onset and severity of the disease in individuals who were exposed to similar levels of cigarette smoke is highly variable, and emerging evidence suggests that the risk of developing COPD is influenced by genetic factors [2], [3]. However, the role of genetic factors and their contribution to disease-causing mechanisms in the *in vivo* pathogenesis of COPD remain poorly understood.

Previous studies in a mouse model with airway-specific overexpression of the β-subunit of the amiloride-sensitive Na^+^ channel (ENaC), which constitutes a limiting pathway for absorption of Na^+^ and fluid across airway epithelia, identified airway surfaces liquid (ASL) dehydration as a disease-causing mechanism of COPD and established a model to study its pathogenesis *in vivo* [4]. Proper regulation of ASL volume by coordinate regulation of ENaC-mediated Na^+^ absorption and Cl^−^ secretion mediated by the cystic fibrosis transmembrane conductance regulator (CFTR) and Ca^2+^-activated Cl^−^ channels (CaCC) plays a crucial role in maintaining normal mucociliary clearance, which constitutes an important innate defense mechanism of the lung [5], [6]. In βENaC-overexpressing (βENaC-Tg) mice, an imbalance between Na^+^ absorption and Cl^−^ secretion results in volume depletion (dehydration) of airway surfaces causing a spontaneous lung disease that shares key features with COPD in humans including impaired mucus clearance, airway mucus obstruction, goblet cell metaplasia, chronic neutrophilic inflammation with increased levels of the IL-8 homologue KC, reduced clearance of bacterial pathogens and emphysema [4], [7], [8]. Of note, a series of recent studies showed that cigarette smoke impairs CFTR-mediated Cl^−^ secretion across airway epithelia *in vitro* and *in vivo* indicating that impaired ASL hydration may also be implicated in the pathogenesis of COPD in humans [9]–[12].

When βENaC-overexpressing (βENaC-Tg) mice were maintained on a mixed genetic background (C3H/He x C57BL/6), we noted that the pulmonary phenotype was highly variable. Around 50% of βENaC-Tg mice died during the neonatal period due to severe mucus plugging of the trachea associated with hypoxic degeneration of airway epithelial cells and asphyxia, whereas the surviving βENaC-Tg mice developed chronic bronchitis and emphysema [4], [13]. These observations suggested that similar to COPD in humans, the COPD-like lung disease in this model may also be modulated by the genetic background.

In the present study, we therefore backcrossed βENaC-Tg mice onto two distinct inbred mouse strains (C57BL/6 and BALB/c) and performed quantitative phenotyping to test the hypothesis that dehydration-induced lung disease can be influenced by the genetic background. Because lung disease in βENaC-Tg mice is caused by a dysbalance between absorption and secretion of NaCl and fluid across airway surfaces, a focus of our studies was on the impact of the genetic background on ENaC-mediated Na^+^ transport and Cl^−^ secretion mediated by CFTR and Ca^2+^−activated Cl^−^ channels (CaCC) in freshly excised airway tissues. Further, we studied the effects of the genetic background on mortality and other characteristic early lesions, i.e. mucus plugging of the trachea, airway epithelial necrosis and inflammation at neonatal ages, and on characteristic features of chronic lung disease including airway mucus obstruction, goblet cell metaplasia, airway inflammation and emphysema formation in surviving βENaC-Tg mice [4], [13]. Because these studies indicated that background-dependent differences in CFTR activity were associated with the severity of neonatal mucus plugging, airway epithelial necrosis and mortality, we also crossed βENaC-Tg mice with gut-corrected CFTR-deficient mice [14] to validate the role of CFTR for the onset and severity of early airways disease.

## Materials and Methods

### Experimental animals

All animal studies were approved by the Animal Care and Use Committee of the Regierungspräsidium Karlsruhe, Germany (approval number 35–9185.81/G-120/05). The βENaC-Tg mouse (line 6608) was originally generated on a mixed genetic background (C3H/He x C57BL/6) [15], [16] and backcrossed to C57BL/6 and BALB/c backgrounds for a minimum of 10 generations. Transgene positive animals were identified by PCR of genomic DNA, as previously described [15]. βENaC-Tg mice on the C57BL/6 and BALB/c background were studied at neonatal (3-day-old) and adult (3-week-old) ages, and wild-type (WT) littermates served as controls in all experiments. Gut corrected CF (CFTR^−/−^) mice overexpressing human CFTR in the intestine under control of the fatty acid binding protein promoter (FABP-hCFTR-CFTR^−/−^) on the FVB background were kindly provided by Dr. Jeffrey A. Whitsett and genotyped, as previously described [14]. The gut-specific overexpression of hCFTR rescues the lethal intestinal phenotype of CF mice thus allowing studies of the effect of CFTR deficiency in the lung independent of concomitant intestinal obstruction [14]. Gut corrected CF mice (FABP-hCFTR-CFTR^−/−^) were intercrossed with βENaC-Tg mice on the C57BL/6 background and double-transgenic βENaC-Tg/CF mice, single-transgenic βENaC-Tg mice, CF mice and WT littermate controls were studied at newborn (PN 0.5) and neonatal (3-day-old) ages. All mice used in this study carried the FABP-hCFTR transgene. Experimental mice were housed in a specific pathogen-free animal facility and had free access to chow and water.

### Electrogenic ion transport measurements

Neonatal (3-day-old) mice were deeply anesthetized via intra-peritoneal injection of a combination of ketamin/xylazin (120 mg/kg and 16 mg/kg, respectively) and killed by exsanguination. Tracheal tissues were dissected and mounted into perfused micro-Ussing chambers with a circular open area of 0.5 mm^2^ [17]. The luminal and basolateral bath was perfused continuously at a rate of ∼10 ml/min, with a solution of the following composition (mM): NaCl 145, KH_2_PO_4_ 0.4, K_2_HPO_4_ 1.6, D-glucose 5, MgCl_2_ 1, Ca-gluconate 1.3, pH 7.4, at 37°C. Experiments were performed under open-circuit conditions as previously described [17], [18]. In brief, values for the transepithelial voltage (V_te_) were referenced to the serosal side. The transepithelial resistance (R_te_) was determined by applying intermittent (1 s) current pulses (ΔI = 0.5 µA) and the equivalent short-circuit current (I_sc_) was calculated according to Ohm's law from V_te_ and R_te_ (I_sc_ = V_te_/R_te_). After an equilibration period of 40 minutes in the Ussing chamber, basal I_sc_ was determined and amiloride (100 μM, luminal) was added to inhibit electrogenic Na^+^ absorption. Then, 3-isobutyl-1-methylxanthine (IBMX; 100 μM, luminal) and forskolin (1 μM, luminal) were added to induce cAMP-mediated Cl^−^ secretion. In some experiments, we also added uridine-5′-triphosphate (UTP) (100 μM, luminal) to induce Ca^2+^-activated Cl^−^ secretion [17]. In additional protocols, tissues were treated which bumetanide (100 µM, basolateral), an inhibitor of the basolateral Na^+^-K^+^-2Cl^−^ cotransporter in the presence of amiloride to block transepithelial Cl^−^ secretion [17]. Further, the CFTR blocker CFTR_inh_-172 (20 μM, luminal) was added to amiloride-pretreated tissues in the absence or presence of cAMP-mediated stimulation (IBMX/forskolin) to determine CFTR-mediated Cl^−^ secretion [19]. Amiloride dose-response curves were obtained by measuring the change in I_sc_ induced by exposing tissues to increasing concentrations of amiloride (10^−9^ to 10^−3^ M, luminal) and plotted as remaining amiloride-sensitive I_sc_ (I) normalized to the maximal amiloride-sensitive I_sc_ (I_max_). IC_50_ values were determined by fitting dose-response data to the Hill equation with the Hill coefficient constrained to 1.0: I_amil_ = I_max_ * [1–1/(1+ IC_50_/*A*)], where *A* is the apical bath amiloride concentration. Amiloride, IBMX, forskolin and bumetanide were obtained from Sigma (Steinheim, Germany), UTP was obtained from GE Healthcare (Buckinghamshire, UK) and the CFTR inhibitor-172 was obtained from Calbiochem (Darmstadt, Germany). All chemicals were of the highest purity grade available.

### Histology and airway morphometry

Anesthetized mice were killed by exsanguination, lungs and tracheae were removed through a median sternotomy, immersion fixed in 4% buffered formalin, and embedded in paraffin. Tracheae were sectioned longitudinally, and lungs were sectioned transversally at the level of the proximal intrapulmonary main axial airway near the hilus. Sections were cut at 5 µm and stained with hematoxylin and eosin (H&E) or alcian blue periodic acid-Schiff (AB-PAS) as previously described [4]. For quantitative assessment of airway mucus obstruction, we used Analysis B image analysis software (Olympus, Hamburg, Germany) to determine mucus volume density as previously described [4]. In brief, images of airway sections were taken with an Olympus IX-71 microscope (Olympus, Hamburg, Germany), the length of the airway boundary, as defined by the epithelial basement membrane, was measured by the interactive image measurement tool and the AB-PAS positive surface area within this boundary was measured by phase analysis according to the automatic threshold settings of the software. The volume density of airway mucus, representing the volume of airway mucus content per surface area of the basement membrane (nl/mm^2^), was determined from the surface area of AB-PAS positive mucus and the basement membrane length, as previously described [20], [21]. Goblet cells were identified by the presence of intracellular AB-PAS positive material and degenerative airway epithelial cells were identified by morphologic criteria (i.e., cell swelling with cytoplasmic vacuolization), and numeric cell densities were quantitated by counting epithelial cells per mm of the basement membrane [4].

### Lung volume and mean linear intercepts

Lungs of 3-week-old mice were inflated with 4% buffered formalin to 25 cm of fixative pressure, and lung volume determined by the volume displacement method [22]. Subsequently, lungs were processed for histology, sectioned at 5 μm, and stained with hematoxylin and eosin (H&E). Histological images were digitally captured with an Olympus IX 71 microscope, using Analysis B image analysis software (Olympus, Hamburg, Germany) with a line counting tool at a magnification of 16x beginning at a randomly selected point. Mean linear intercepts were determined by dividing the sum of the lengths of all lines in all frames by the number of intercepts between alveolar septi and counting lines, as previously described [4], [23]. For each animal, we measured a minimum of 200 intercepts sampled in 10 fields in different lobes.

### Real-time RT-PCR

Tracheal tissues were immediately stored in RNA later (Applied Biosystems, Darmstadt, Germany). RNA was isolated by using Trizol reagent (Invitrogen, Karlsruhe Germany) and RNeasy Mini Kit (Qiagen, Hilden, Germany). RNA from tracheae of four neonatal mice of the same genotype were pooled, RNA purity and quantity was determined using a NanoDrop ND100 spectrophotometer (PeqLab, Erlangen, Germany) and integrity was verified by with Agilent 2100 Bioanalyzer (Agilent Technologies, Santa Clara, CA, USA). cDNA was obtained by reverse transcription of 1 μg of total RNA (Superscript III RT; Invitrogen, Karlsruhe, Germany). Quantitative real-time RT-PCR for αENaC, βENaC, γENaC, CFTR, Muc5 b and glyceraldehyde 3-phosphate dehydrogenase (Gapdh) was performed on an Applied Biosystems 7500 Real Time PCR System using TaqMan universal PCR master mix and inventoried TaqMan gene expression assays according to the manufacturer's instructions (Applied Biosystems, Darmstadt, Germany). Relative fold changes in target gene expression were determined from the efficiency of the PCR reaction and the crossing point deviation between WT and βENaC-Tg from the C57BL/6 and BALB/c backgrounds and normalized to the expression of the reference gene Gapdh, as previously described [4], [24].

### BAL cell counts and cytokine measurements

Adult mice were deeply anesthetized, the trachea cannulated, and the lung lavaged with PBS. Bronchoalveolar lavage (BAL) samples were centrifuged, total cell counts were determined in a hemocytometer and differential cell counts were determined on cytospin preparations stained with May-Grünwald-Giemsa, as previously described [4], [24]. Concentrations of KC and TNF-α in neonatal mouse lungs were measured in lung homogenates using ELISA (R&D Systems, Minneapolis, MN) according to manufacturer's instructions. In briefly, neonatal lungs were homogenized in 250 μl of PBS containing protease inhibitors (Roche, Mannheim, Germany), homogenates were centrifuged and the cell-free supernatant was used for ELISA [4].

### Statistics

All experiments were performed by an investigator blinded to the genotype of the mice. Data were analysed with SigmaStat version 3.1 (Systat Software, Erkrath, Germany) and are reported as mean ± SEM. Statistical analyses were performed using unpaired Student's *t-*test, Mann-Whitney Rank Sum test, Chi-square test, Kaplan-Meier survival analysis, as appropriate and *P*<0.05 was accepted to indicate statistical significance.

## Results

### Survival of βENaC-Tg mice is modified by genetic background

**Figure 1:**
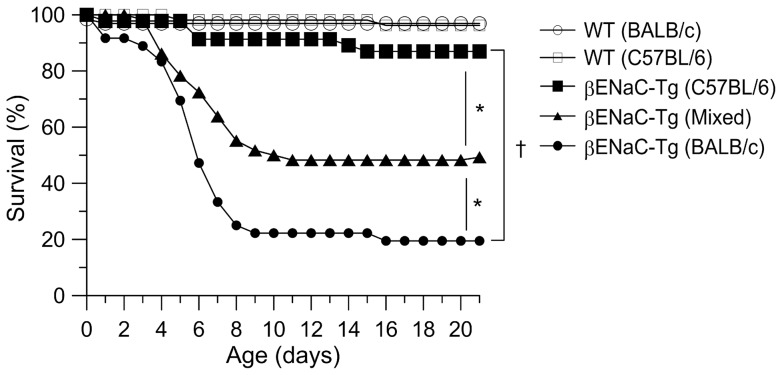
Survival of βENaC-Tg mice is modified by genetic background. Survival curves of βENaC-Tg mice and wild-type (WT) littermates on mixed (C3H/He x C57BL/6), C57BL/6 and BALB/c backgrounds (n = 36–59 mice per group). *P<0.05 and ^†^ *P*<0.01 (Kaplan-Meier survival analysis).

On a mixed genetic background (C3H/HeN x C57BL/6), around 50% of βENaC-Tg mice died due to severe airway mucus obstruction in the first weeks of life, whereas the remaining ∼50% showed normal survival but developed chronic obstructive lung disease [4], [13], [15]. We speculated that this variability in disease severity may be determined by modifiers in the genetic background. We therefore backcrossed βENaC-Tg mice with the original mixed background to the inbred C57BL/6 and BALB/c backgrounds, respectively (Fig. 1). Compared to the mixed background, backcross to C57BL/6 significantly reduced mortality of βENaC-Tg mice to 14% (*n* = 46, *P*<0.05), whereas backcross to the BALB/c background significantly increased mortality to 81% (*n* = 36, *P*<0.05). These results indicate that mortality of βENaC-Tg mice is modulated by the genetic background.

### Genetic background modulates airway ion transport in WT and βENaC-Tg mice

**Figure 2:**
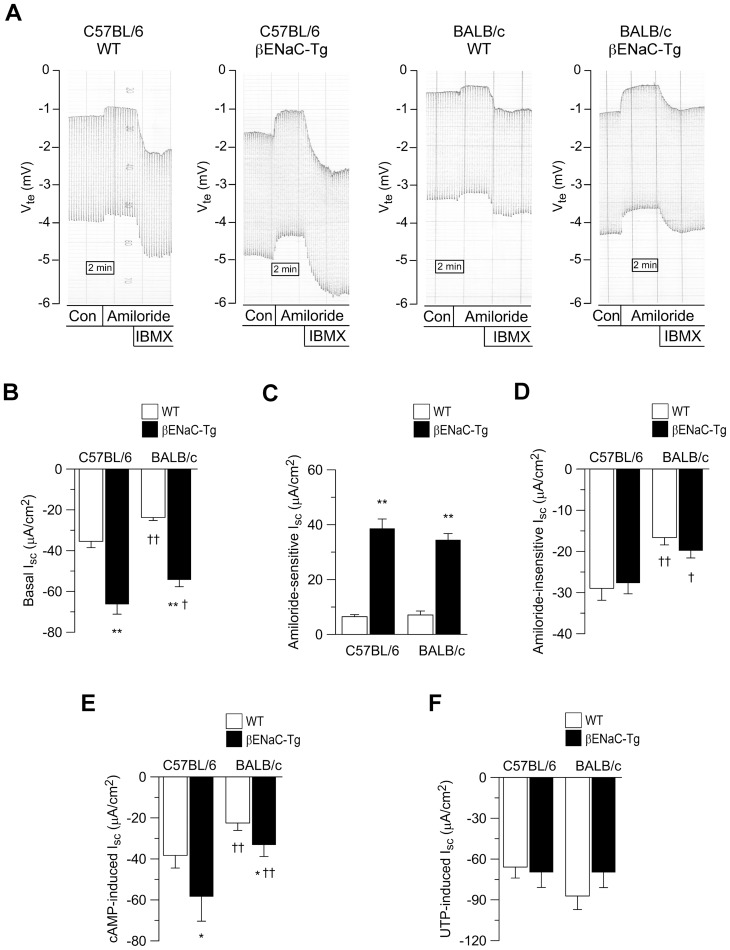
Genetic background modulates airway ion transport in wild-type and βENaC-Tg mice. A) Representative original recordings of the effects of amiloride and cAMP-dependent activation (IBMX/forskolin) on transepithelial voltage (V_te_) and transepithelial resistance (R_te_) across freshly excised tracheal tissues from neonatal (3-day-old) wild-type (WT) and βENaC-Tg mice on the C57BL/6 and BALB/c background. R_te_ was determined from V_te_ deflections obtained by pulsed current injection. (B–F) Summary of basal equivalent short-circuit current (I_sc_) (B), amiloride-sensitive I_sc_ (C), amiloride-insensitive I_sc_ (D), cAMP-induced I_sc_ (E) and UTP-induced I_sc_ (F) in freshly excised tracheal tissues from neonatal WT and βENaC-Tg mice on the C57BL/6 and BALB/c background. Data are presented as mean ± SEM (*n* = 8–20 mice per group). *P<0.05 and **P<0.001 compared with WT mice on same strain background, ^†^ *P*<0.05 and ^††^ *P*<0.01 compared with mice of same genotype on C57BL/6 background.

Early death in βENaC-Tg mice is caused by a dysbalance of airway Na^+^ absorption and Cl^−^ secretion producing ASL volume depletion and airway mucus plugging [15]. We therefore hypothesized that the background-dependent differences in mortality may be related to differences in airway epithelial Na^+^ and/or Cl^−^ transport in neonatal βENaC-Tg mice on the C57BL/6 *versus* BALB/c backgrounds. To test this hypothesis, we determined basal bioelectric properties, amiloride-sensitive Na^+^ absorption, and cAMP-induced and Ca^2+^−activated (UTP-mediated) Cl^−^ secretion in freshly excised tracheal tissues from 3-day-old neonatal βENaC-Tg mice on the C57BL/6 and BALB/c backgrounds and their respective WT littermates (Fig. 2).

Similar to previous results on the mixed background, the comparison between WT and βENaC-Tg tissues within each inbred background demonstrated that overexpression of βENaC significantly increased basal and amiloride-sensitive I_sc_ in both C57BL/6 and BALB/c mice (*P*<0.001) (Fig. 2A–C). Further, the cAMP-induced I_sc_ was increased and the UTP-mediated I_sc_ remained unchanged in βENaC-Tg tissues from both backgrounds compared to their respective WT tissues (Fig. 2A,E,F). Of note, the comparison between the two inbred backgrounds (i.e. C57BL/6 *versus* BALB/c) identified substantial effects of the genetic background on several components of airway ion transport. Specifically, the basal I_sc_, amiloride-insensitive I_sc_ and cAMP-induced I_sc_ were significantly greater in WT tissues from the C57BL/6 background compared to the BALB/c background (*P*<0.01 to *P*<0.001) (Fig. 2A,B,D,E). Similar background-dependent differences were observed for βENaC-Tg tissues (*P*<0.05 to *P*<0.01) (Fig. 2A,B,D,E). These results suggest that the C57BL/6 background is associated with higher levels of basal and cAMP-induced Cl^−^ secretion in both WT and βENaC-Tg airways.

Conversely, the genetic background had no effect on the magnitude of amiloride-sensitive Na^+^ transport in airways from WT or βENaC-Tg mice (Fig. 2C). Because previous studies indicated that overexpression of βENaC produces a subpopulation of αβENaC channels that are less sensitive to amiloride and physiological ENaC inhibitors (including endogenous anti-proteases) than normal αβγENaC channels, and that these dysregulated αβENaC channels may therefore aggravate ASL depletion under physiological “thin film” conditions *in vivo* [25], [26], we also determined transcript levels of α-, β-, and γENaC subunits and amiloride sensitivity in WT and βENaC-Tg tracheal tissues from the C57BL/6 and BALB/c backgrounds. In βENaC-Tg airways from both backgrounds, βENaC mRNA was overexpressed to similar levels (Fig. S1A-C), which produced a similar decrease in amiloride sensitivity, as determined from similar shifts of the amiloride dose-response curves and IC_50_ values on both backgrounds (Fig. S1D-F). Collectively, these results indicate that the genetic background had no effect on the magnitude or regulation of increased airway Na^+^ transport in βENaC-Tg mice.

### Genetic background modulates CFTR-mediated Cl^−^ secretion in airways of WT and βENaC-Tg mice

**Figure 3 pone-0044059-g003:**
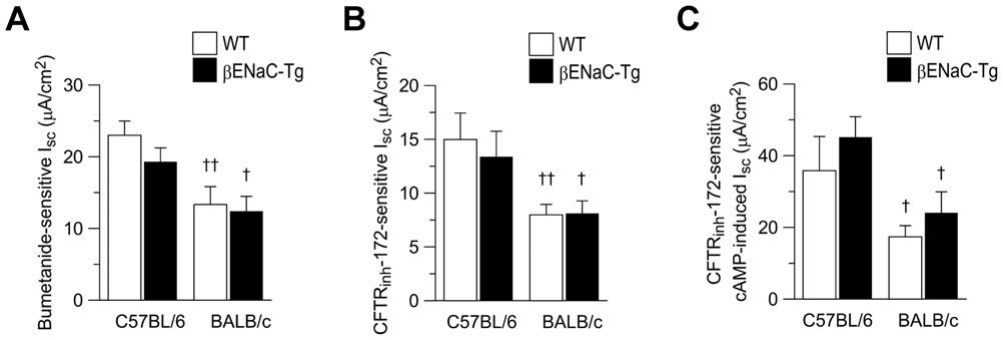
Genetic background modulates CFTR-mediated Cl^−^ secretion in airways of wild-type and βENaC-Tg mice. A–C) Effects of genetic background on transepithelial Cl- secretion were determined by adding bumetanide or CFTR_inh_-172 to amiloride-pretreated tracheal tissues from neonatal wild-type (WT) and βENaC-Tg mice on the C57BL/6 and BALB/c background. A,B) Summary of bumetanide-sensitive I_sc_ (A) and CFTR_inh_-172-sensitive I_sc_ (B) in the presence of amiloride (*n* = 12–23 mice per group). C) Summary of CFTR_inh_-172-sensitive I_sc_ in the presence of amiloride and cAMP-dependent activation (IBMX/forskolin) (*n* = 5–11 mice per group). Data are presented as mean ± SEM. ^†^ *P*<0.05 and ^††^ *P*<0.01 compared with mice of same genotype on C57BL/6 background.

To obtain a more detailed characterization of the origin of the increased amiloride-insensitive I_sc_ observed in tracheal tissues of neonatal C57BL/6 WT and βENaC-Tg mice compared to BALB/c mice of the same genotype, we perfused tissues with bumetanide to block transepithelial Cl^−^ secretion or CFTR_inh_-172 to probe for CFTR activity. Similar to previous studies in adult BALB/c WT mice [17], the amiloride-insensitive I_sc_ was largely abolished by bumetanide in neonatal WT and βENaC-Tg tissues from both backgrounds (Fig. 2D and Fig. 3A) demonstrating that this residual current reflected basal Cl^−^ secretion. Of note, the bumetanide-sensitive I_sc_ was significantly increased in tracheal tissues from WT and βENaC-Tg mice on the C57BL/6 compared to the BALB/c background (*P*<0.05 and *P*<0.01) (Fig. 3A) demonstrating that basal Cl^−^ secretion was greater on the C57BL/6 background independent of the genotype. Consistent with previous studies in neonatal CFTR-deficient mice [27], we show that ∼50% of the amiloride-insensitive I_sc_ were inhibited by CFTR_inh_-172 (Fig. 2D and Fig. 3B) indicating that CFTR contributes to basal Cl^−^ secretion in the neonatal trachea. Similar to bumetanide, the CFTR_inh_-172-sensitive I_sc_ was significantly increased in tracheal tissues from WT and βENaC-Tg mice on the C57BL/6 compared to the BALB/c background *P*<0.05 to *P*<0.01).

Because cAMP-induced Cl^-^ secretory responses were also increased in airways from C57BL/6 compared to BALB/c mice (Fig. 2E), we next determined the effects of CFTR_inh_-172 in the presence of cAMP-dependent stimulation. The cAMP-induced I_sc_ was largely abolished by CFTR_inh_-172 in WT and βENaC-Tg mice on both backgrounds (Fig. 2E and Fig. 3C). Interestingly, the magnitude of CFTR_inh_-172-sensitive I_sc_ in the presence of cAMP-mediated activation was significantly increased in airways from WT and βENaC-Tg mice on the C57BL/6 compared to the BALB/c background (*P*<0.05). Taken together, these results demonstrate that CFTR-mediated Cl^−^ secretion is increased in native airway tissues from neonatal C57BL/6 compared with BALB/c mice and suggest that higher levels of Cl^−^ secretion may counteract ASL depletion due to increased Na^+^ absorption and thus improve mucus clearance in βENaC-Tg mice [4], [13], [15].

To determine, if strain-dependent differences in CFTR-mediated Cl^−^ conductance were caused by differences in CFTR transcript levels, we next compared CFTR mRNA expression in freshly excised tracheal tissues in WT and βENaC-Tg mice on both backgrounds. These studies showed that the genetic background had no effect on relative CFTR mRNA levels in either WT mice (1.0±0.10 for WT C57BL/6 mice versus 0.96±0.12 for WT BALB/c mice; n = 6 per group; *P* = 0.91) or βENaC-Tg mice (1.0±0.08 for βENaC-Tg C57BL/6 versus 1.03±0.16 for βENaC-Tg BALB/c; *n* = 6 per group; *P* = 0.87) suggesting that the strain-dependent differences in CFTR-mediated Cl^−^ transport reflect differences in post-transcriptional regulation of CFTR in murine airways.

### Genetic background modifies early airway mucus obstruction and epithelial necrosis but not inflammation in neonatal βENaC-Tg mice

**Figure 4:**
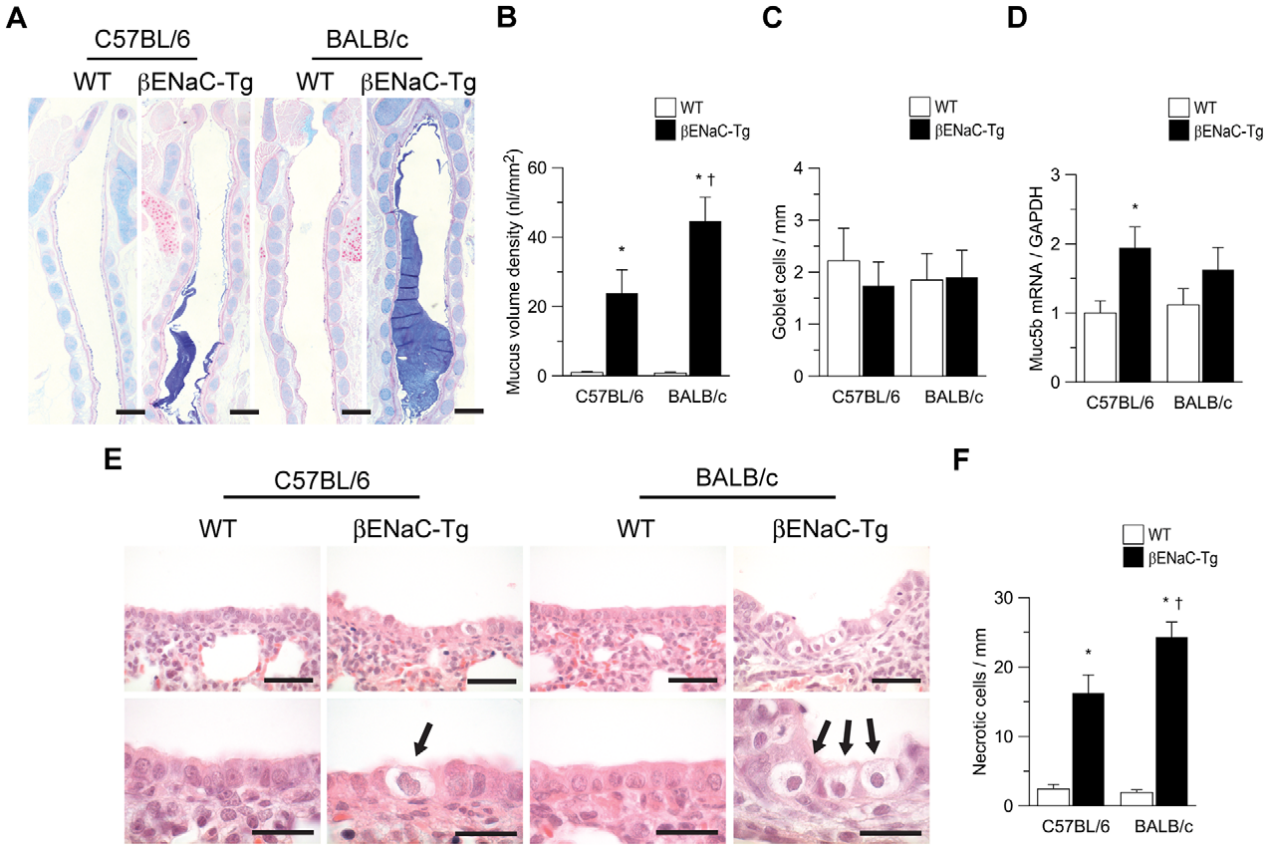
Genetic background modifies early airway mucus obstruction and epithelial necrosis in neonatal βENaC-Tg mice. A) Longitudinal sections of tracheae from neonatal (3-day-old) wild-type (WT) and βENaC-Tg mice on the C57BL/6 and BALB/c background. Sections were stained with Alcian blue–periodic acid Schiff (AB-PAS) to determine the presence of mucus and goblet cells. Scale bars  = 200 µm. B–D) Summary of mucus content, as determined from measuring volume density of AB-PAS-positive material in the tracheal lumen (B), goblet cell numbers (C) and Transcript levels of Muc5b (D) (*n* = 4–13 mice per group). E) Airway histology from neonatal (3-day-old) WT and βENaC-Tg mice on the C57BL/6 and BALB/c background. Sections were stained with hematoxylin and eosin (H&E) and evaluated for degenerative airway epithelial cells (arrows). Scale bars  = 40 µm (upper panels) and 20 µm (lower panels). F) Summary of airway epithelial necrosis as determined from the number of degenerative epithelial cells per mm of the basement membrane (*n* = 9–11 mice per group). *P<0.001 compared with WT mice on same strain background, ^†^ *P*<0.05 compared with mice of same genotype on C57BL/6 background.

To determine the relationship between background-dependent differences in airway Cl^−^ secretion and early mucus plugging, we next compared the extent of intraluminal mucus obstruction and transcript levels of the airway mucin Muc5b in lungs from 3-day-old neonatal βENaC-Tg mice on the C57BL/6 and BALB/c backgrounds. Similar to previous studies on the mixed genetic background [4], [13], mucus obstruction was readily detected in the trachea, but not in intrapulmonary airways of neonatal βENaC-Tg mice on both backgrounds (Fig. 4A,B and data not shown). Morphometric analyses of tracheal sections demonstrated that mucus content was significantly decreased (∼2-fold) in βENaC-Tg mice on the C57BL/6 compared to the BALB/c background (*P*<0.05). These differences in tracheal mucus content were not associated with differences in goblet cell densities or levels of Muc5b mRNA expression in the lung. These results indicate that distinct levels of mucus obstruction in βENaC-Tg C57BL/6 *versus* βENaC-Tg BALB/c mice did not reflect background-related differences in mucus production, as estimated from Muc5b transcript levels and the numbers of mucin producing cells (Fig. 4A–D).

In addition to airway mucus obstruction, unregulated Na^+^ absorption was shown to produce cellular hypoxia, hydropic degeneration and necrosis of airway epithelial cells constituting another characteristic lesion in neonatal βENaC-Tg mice [4], [26]. We, therefore, compared the extent of epithelial necrosis in 3-day-old βENaC-Tg mice on the C57BL/6 compared to the BALB/c background (Fig. 4E,F). Necrotic airway cells were readily detected in βENaC-Tg mice from both backgrounds, however, the number of necrotic cells was significantly decreased on the C57BL/6 compared to the BALB/c background (Fig. 4F).

Previous studies in βENaC-Tg mice on a mixed genetic background demonstrated that early airway mucus obstruction and epithelial necrosis were associated with the onset of airway inflammation [4]. Accordingly, expression of the pro-inflammatory cytokines KC and TNF-α was also increased in lung homogenates from βENaC-Tg compared to WT neonates on the C57BL/6 as well as BALB/c background (Fig. S2A,B). In contrast to mucus obstruction and epithelial necrosis (Fig. 4), levels of KC and TNF-α were not different in βENaC-Tg mice on the C57BL/6 compared to the BALB/c background (Fig. S2A,B). Taken together, these results indicate that higher levels of endogenous CFTR-mediated Cl^−^ secretion on the C57BL/6 background counteracted some of the early consequences of Na^+^ hyperabsorption and reduced early death due to airway mucus plugging in βENaC-Tg mice. Conversely, our results also suggest that the lower levels of airway mucus obstruction and necrosis observed in βENaC-Tg C57BL/6 mice were sufficient to trigger inflammation to a similar level as observed in βENaC-Tg BALB/c mice.

### Genetic background has no effect on chronic obstructive lung disease in adult βENaC-Tg mice

To determine, if the genetic background also affected the severity of chronic lung disease, we next compared the extent of airway mucus obstruction and goblet cell metaplasia in intrapulmonary airways, inflammation and emphysema characteristic of in 3-week-old surviving βENaC-Tg mice on the C57BL/6 and BALB/c backgrounds [4] (Fig. S2). Several differences including densities of airway goblet cells and BAL leukocyte counts were observed in WT C57BL/6 compared to WT BALB/c mice (Fig. S2B,C,G). However, the extent of airway mucus obstruction and goblet cell metaplasia (Fig. S2A-C), emphysema formation as determined from measurements of mean linear intercepts and lung volume (Fig. S2D-F), and elevated BAL inflammatory cell counts did not differ in βENaC-Tg C57BL/6 *versus* βENaC-Tg BALB/c mice (Fig. S2G). These results indicate that background-related differences in CFTR-mediated Cl^−^ secretion improved neonatal survival, but had no impact on the severity of chronic lung disease in βENaC-Tg mice.

### Lack of CFTR increases early airway mucus obstruction, airway epithelial necrosis and mortality in βENaC-Tg mice

**Figure 5:**
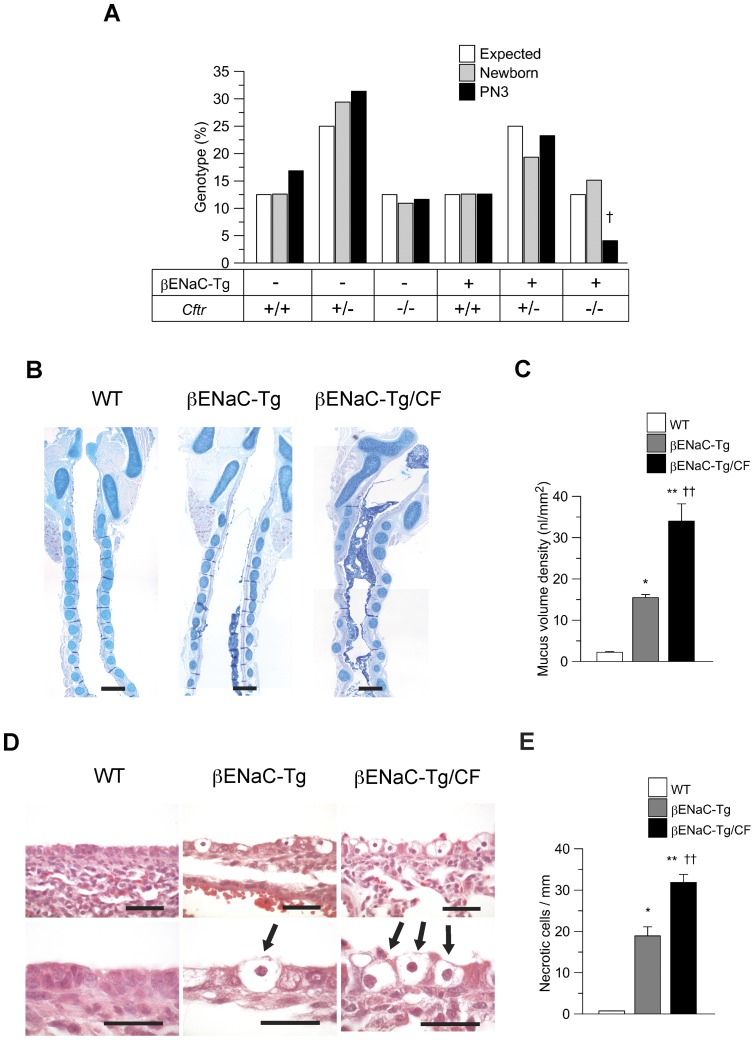
Lack of CFTR increases early airway mucus obstruction, epithelial necrosis and mortality in βENaC-Tg mice. A) Neonatal mortality in double-mutant βENaC-Tg/CF mice, single-transgenic βENaC-Tg mice, CF mice and wild-type (WT) littermate controls was determined from the distribution of genotypes in newborn (PN 0.5) and 3-day-old pups from the intercross of βENaC-Tg and CF mice (*n* = 119–252 mice per age group). ^†^ *P*<0.05 compared with newborn mice of same genotype. B) Longitudinal sections of tracheae from newborn (PN 0.5) WT, single-transgenic βENaC-Tg and double-mutant βENaC-Tg/CF mice. Sections were stained with Alcian blue–periodic acid Schiff (AB-PAS) to determine the presence of intraluminal mucus. Scale bars  = 200 µm. C) Summary of mucus content, as determined from measuring volume density of AB-PAS-positive material in the lumen (*n* = 4–6 mice per group). D) Airway histology from neonatal (3-day-old) WT, βENaC-Tg and double-mutant βENaC-Tg/CF mice. Sections were stained with hematoxylin and eosin (H&E) and evaluated for degenerative airway epithelial cells (arrows). Scale bars  = 40 µm (upper panels) and 20 µm (lower panels). E) Summary of airway epithelial necrosis as determined from the number of degenerative epithelial cells per mm of the basement membrane (*n* = 4–10 mice per group). *P<0.01 and **P<0.001compared with WT, ^††^ *P*<0.01 compared with βENaC-Tg mice.

For independent validation of the role of CFTR as a modifier of early airway disease in βENaC-Tg mice, we next crossed βENaC-Tg mice with gut-corrected CF mice [14] and compared survival, early mucus obstruction and airway epithelial necrosis in neonatal single-transgenic βENaC-Tg mice and double-mutant βENaC-Tg/CF mice (Fig. 5). Gut-corrected CF mice were used for these studies to determine effects on pulmonary mortality independent of concomitant mortality due to intestinal obstruction [14]. While the onset of mortality in single-transgenic βENaC-Tg mice typically begins at ∼3 days of age, initial studies indicated that most double-mutant βENaC-Tg/CF mice died even earlier and escaped analyses because the carcasses were eaten by their dams. To obtain more accurate estimates of neonatal mortality, we therefore genotyped all remaining offspring from the intercross either on the day of birth (PN 0.5) or at the age of 3 days, and compared the frequencies of genotypes at the two time points. At birth, all genotypes were represented at the expected Mendelian ratios. At the age of 3 days, the frequency of alive single-transgenic βENaC-Tg mice and CF mice remained unchanged, whereas the frequency of surviving βENaC-Tg/CF mice was significantly reduced by ∼70% (*P*<0.05) (Fig. 5A). Morphometric analyses of tracheal sections from neonatal (PN 0.5) mice demonstrated that intraluminal mucus obstruction was significantly increased in βENaC-Tg/CF mice compared to single-transgenic βENaC-Tg (*P*<0.01) littermates (Fig. 5B,C). Further, the numeric densities of necrotic airway cells were significantly increased in βENaC-Tg/CF versus βENaC-Tg mice (Fig. 5D,E). Similar to WT mice, CF mice did not show mucus obstruction or epithelial cell necrosis (data not shown). Levels of the pro-inflammatory cytokine KC were not different in lungs of newborn βENaC-Tg and βENaC-Tg/CF mice (Fig. S4A). In both groups, KC concentrations tended to be higher than in WT and CF mice, but this difference did not reach statistical significance. Similar, levels of TNF-α were neither elevated in newborn CF, nor in βENaC-Tg or double-mutant βENaC-Tg/CF mice compared to WT littermates (Fig. S4B). Collectively, these data demonstrate that lack of CFTR aggravates tracheal mucus obstruction, airway epithelial necrosis and mortality providing independent genetic evidence that CFTR modifies the onset of airway disease in βENaC-Tg mice.

## Discussion

This study demonstrates that the genetic background has a substantial influence on CFTR activity in murine airways, and that genetically determined differences in CFTR function have profound effects on the severity of dehydration-induced lung disease in βENaC-Tg mice. In the airways, CFTR acts as a cAMP-dependent Cl^−^ channel as well as a regulator of ENaC, and plays an important role in the regulation of proper airway surface hydration that is essential for effective mucociliary clearance [5], [6], [18], [28], [29]. In βENaC-Tg mice, increased ENaC-mediated Na^+^ absorption and airway surface dehydration were associated with reduced mucus clearance and spontaneous airway mucus plugging resulting in neonatal mortality in a subset of βENaC-Tg mice [4], [15], [30]. Further, recent studies identified hydropic degeneration and necrosis of βENaC-Tg-expressing airways cells, likely triggered by cellular hypoxia caused by reduced oxygen tension due to airway mucus plugging, combined with increased oxygen demands due to excessive Na^+^ entry into cells, as another characteristic early lesion in βENaC-Tg mice [4], [26]. Our bioelectric studies in native airway tissues demonstrated that endogenous CFTR-mediated Cl^−^ secretion was ∼50% decreased in both WT and βENaC-Tg mice on the BALB/c compared to the C57BL/6 background (Figs. 2 and 3). In contrast, the magnitude of CaCC-mediated Cl^−^ secretion and ENaC-mediated Na^+^ absorption constituting other pathways critical for ASL regulation [26], [27], were not influenced by the genetic background (Fig. 2 and Fig. S1). Decreased levels of CFTR function on the BALB/c background had no effects on airway morphology in WT mice, but were associated with strikingly more severe tracheal mucus plugging, airway epithelial necrosis and mortality in βENaC-Tg mice (Figs. 1 and 4).

The importance of CFTR function in early airways disease was validated in independent studies, in which βENaC-Tg mice were crossed with CFTR-deficient mice. These studies demonstrated that neonatal mucus obstruction, epithelial necrosis and mortality were substantially increased, when CFTR was genetically deleted in double-mutant βENaC-Tg/CF mice compared to single transgenic βENaC-Tg mice (Fig. 5). Interestingly, lack of CFTR enhanced these airway pathologies prior to the onset of inflammation, as determined from measurements of the pro-inflammatory cytokines KC and TNF-α in lungs from newborn mice (Fig. S4). These results are consistent with an important role of CFTR in the regulation of ENaC function in airway epithelia *in vivo*, as previously demonstrated in heterologous cells and airway epithelial cell lines *in vitro* [28], [31]–[33]. Of note, a recent study demonstrated that overexpression of human CFTR (hCFTR) failed to ameliorate lung disease in βENaC-Tg mice [34], probably due to different chromosomal integration sites of the transgenic constructs after pronuclear injection leading to expression of hCFTR and βENaC transgenes in different subpopulations of Clara cells [35], or because hCFTR din not function properly in the context of mouse Clara cells [36]. In contrast to this transgenic approach based on overexpression of human CFTR [34], our studies relied on naturally occurring variability and knockout of endogenously expressed murine CFTR. Collectively, the results from our studies of βENaC-Tg mice on different genetic backgrounds and the cross with CFTR-deficient mice indicate that genetically determined variability of endogenous CFTR function modulates the extent of ASL dehydration and that low levels or absence of CFTR-mediated Cl^−^ secretion aggravate the severity of early airway lesions and related pulmonary mortality in neonatal βENaC-Tg mice.

Conversely, background-dependent differences in CFTR function (Figs. 2 and 3) had no effect on the severity of COPD-like lung disease in surviving βENaC-Tg mice (Fig. S3). These results, together with the observation that expression of the pro-inflammatory cytokines KC and TNF-α were elevated to similar levels in βENaC-Tg mice on both genetic backgrounds as early as 3 days of age (Fig. S2), suggests that the endogenous difference in CFTR activity (∼2-fold) between airway tissues from C57BL/6 and BALB/c mice was not sufficient to prevent long-term consequences and secondary pathologies, including airway inflammation, mucus hypersecretion, goblet cell metaplasia and emphysema triggered by airway surface dehydration in βENaC-Tg mice *in vivo* [4], [13].

While it is well established that impaired CFTR-mediated Cl^−^ secretion and airway surface dehydration due to mutations in the *CFTR* gene cause cystic fibrosis (CF) with early onset and severe chronic obstructive airways disease [37]–[39], little is known about the relationship between endogenous variability of WT CFTR function and susceptibility for lung disease in humans. Interestingly, nasal potential difference (nPD) measurements in healthy non-smokers documented substantial (up to ∼3-fold) inter-individual differences in the magnitude of CFTR-mediated Cl^−^ secretion [10], [40]. These studies indicate that similar to our findings in inbred mouse strains, CFTR activity is also modulated by the genetic background in humans. Interestingly, a series of recent studies demonstrated that cigarette smoke, i.e. a common environmental exposure, has acute effects on CFTR activity in airway epithelia of healthy non-smokers causing ∼60% inhibition of CFTR function *in vivo*, and causes airway surface dehydration and reduced mucus transport *in vitro* [10]–[12]. Further, emerging evidence suggests that smokers who developed COPD exhibit reduced CFTR-mediated Cl^−^ secretion and airway mucus dehydration. [11]. These results indicate that reduced CFTR function may be insufficient for proper mucus hydration in the presence of concomitant cigarette smoke-induced goblet cell metaplasia and mucin hypersecretion [8], [41], and may thus set smokers at risk for mucus stasis and airways disease [7]. Our studies in mice indicate that a similar reduction (∼50%) in endogenous CFTR activity on the BALB/c compared to the C57BL/6 background has indeed a significant impact on the development of mucus obstruction in the airways of βENaC-Tg mice, where mucus is concentrated by increased ENaC-mediated Na^+^ and fluid absorption, as indexed by a substantial increase in the percent solids content of the ASL [15] (Figs. 2 to [Fig pone-0044059-g003] 4). In addition, recent reports demonstrated an inverse relationship between CFTR expression, ceramide accumulation and severity of emphysema in lung tissues from patients with COPD [42]. Further, studies in mice showed that cigarette-smoke decreased CFTR expression in lipid-rafts and demonstrated that CFTR plays an important role in the regulation of apoptotic and autophagic responses in cigarette-smoke induced lung epithelial injury [43]. These studies suggest that reduced CFTR levels in smokers may cause cellular dysfunctions, e.g. altered ceramide metabolism, that may play an important role in the pathogenesis of COPD independent of impaired epithelial Cl^−^ secretion and ASL homeostasis [44], [45]. When viewed in combination, it is tempting to speculate that low levels of endogenous CFTR activity, in addition to other genetic and environmental factors [2], [3], may constitute an important risk factor that makes smokers susceptible for developing COPD. However, future studies are required to determine the relationship between endogenous levels of CFTR-mediated Cl^−^ secretion, exposure to cigarette smoke and other environmental stimuli, and the risk for developing COPD.

In this context, it is noteworthy that a small molecule CFTR modulator, VX-770, recently developed to restore Cl^−^ channel function of mutant CFTR in patients with CF, was also shown to improve the activity of WT CFTR Cl^−^ channels [46] and restore cigarette-smoke induced impairment of CFTR-mediated Cl^−^ secretion, ASL homeostasis and mucus transport in cultured non-CF human bronchial epithelia *in vitro* [12]. In patients with CF with a specific *CFTR* mutation (G551D) that impairs gating of the CFTR Cl^−^ channel, treatment with VX-770 partially restored CFTR-mediated Cl^−^ secretion in nasal epithelia and reduced airflow obstruction and pulmonary exacerbations related to CFTR dysfunction [47], [48]. If future studies in humans can confirm the results from our murine studies demonstrating an inverse relationship between WT CFTR function and severity of obstructive airway disease, VX-770 and potentially other CFTR modulators that improve surface expression and/or function of WT CFTR Cl^−^ channels [49] may provide therapeutic opportunities for COPD in a subgroup of individuals with low levels of endogenous CFTR activity produced by either genetic factors or environmental factors such as cigarette smoke [12]. However, our results in neonatal and adult βENaC-overexpressing mice on different genetic backgrounds (C57BL/6, BALB/c and CFTR^−/−^) (Fig. 4,5 and Fig. S3) also indicate that pharmacological augmentation of CFTR function may be more effective in the early pathogenesis, and that late treatment may not be able to correct or revert established COPD with chronic mucus hypersecretion, airways inflammation and emphysema.

Our phenotype-driven studies did not allow us to identify the mechanisms underlying increased CFTR activity in airways from C57BL/6 compared to BALB/c mice. Analyses of CFTR mRNA expression by real-time RT-PCR demonstrated that CFTR transcript levels were not different in WT or βENaC-Tg airway tissues from C57BL/6 *versus* BALB/c mice suggesting that strain-dependent differences in CFTR-mediated Cl^−^ secretion reflected differences in post-transcriptional and/or post-translational regulation of CFTR processing, trafficking, protein lifetime, or autocrine signalling that regulates CFTR activity at the apical plasma membrane. We expect that the backcross of the βENaC-Tg mouse onto distinct isogenic backgrounds performed in this study, will facilitate the use of genomics approaches such as analyses of quantitative trait loci or whole genome sequencing [50] to identify the molecular mechanisms underlying the genetically determined differences in CFTR activity, and potentially identify other modifiers that may contribute to the striking differences in the neonatal airways phenotype of βENaC-Tg mice on the C57BL/6 and BALB/c backgrounds.

In summary, our studies demonstrate that low levels or absence of CFTR-mediated Cl^−^ secretion aggravate early airway mucus obstruction and pulmonary mortality associated with COPD-like lung disease in βENaC-Tg mice. These results suggest that genetic or environmental factors that modify CFTR function may modulate the onset and severity of airway mucus obstruction and that CFTR may serve as a potential therapeutic target in patients with COPD.

## Supporting Information

Figure S1 **Genetic background has no effect on α, β and γENaC expression and ENaC-mediated Na^+^ transport in airways of wild-type and βENaC-Tg mice.** A–C) Transcript levels of αENaC (A), βENaC (B) and γENaC (C) in freshly excised tracheal tissues from neonatal wild-type (WT) and βENaC-Tg mice on the C57BL/6 and BALB/c background. Data are expressed as fold changes from WT mice on the C57BL/6 background (*n* = 4–5 samples pooled from 16–20 mice per group). D–F) Amiloride dose-response curves (D,E) and summary of IC_50_ values (F) obtained from tracheal tissues of neonatal WT and βENaC-Tg mice on the C57BL/6 and BALB/c background (n = 6–9 mice per group). Data are presented as mean ± SEM. *P<0.01 and **P<0.001 compared with WT mice on same strain background, ^†^ *P*<0.05 compared with mice of same genotype on C57BL/6 background.
(TIF)

Figure S2 **Genetic background has no effect on early airway inflammation in neonatal βENaC-Tg mice.** A, B) Levels of KC (A) and TNF-α (B) in lung homogenates from 3-day-old neonatal wild-type (WT) and βENaC-Tg mice on the C57BL/6 and BALB/c background (*n* = 5–10 mice per group). Data are presented as mean ± SEM. *P<0.05 compared with WT mice on same strain background.
(TIF)

Figure S3 **Genetic background has no effect on chronic lung disease in adult βENaC-Tg mice.** A) Representative airway morphology of adult (3-week-old) wild-type (WT) and βENaC-Tg mice on the C57BL/6 and BALB/c background. Sections were stained with Alcian blue-periodic acid Schiff (AB-PAS) to determine the presence of airway mucus and goblet cells. Scale bars  = 200 µm. B,C) Summary of mucus content, as determined from measuring volume density of AB-PAS-positive material in the airway lumen (B), and goblet cell numbers (C) (*n* = 9–20 mice per group). D) Representative morphology of distal airspaces. Sections were stained with hematoxylin and eosin (H&E). Scale bars  = 100 µm. E–G) Summary of mean linear intercepts (E), lung volume (F), and total and differential cell counts in bronchoalveolar lavage (BAL) fluid (G) from adult WT and βENaC-Tg mice on the C57BL/6 and BALB/c background (*n* = 5–18 mice per group). Data are presented as mean ± SEM. *P<0.05 and **P<0.001 compared with WT mice on same strain background, ^†^ *P*<0.001 compared with mice of same genotype on C57BL/6 background.
(TIF)

Figure S4 **Lack of CFTR does not cause airway inflammation in neonatal mice.** A, B) Levels of KC (A) and TNF-α (B) in lung homogenates from newborn (PN 0.5) wild-type (WT), βENaC-Tg, CFTR-deficient (CF) and double mutant βENaC-Tg/CF mice (*n* = 5–8 mice per group). Data are presented as mean ± SEM. No significant differences were detected between the four experimental groups.
(TIF)

## References

[1] Rabe KF, Hurd S, Anzueto A, Barnes PJ, Buist SA, et al (2007) Global strategy for the diagnosis, management, and prevention of chronic obstructive pulmonary disease: GOLD executive summary. Am J Respir Crit Care Med 176: 532–555. 17507545 10.1164/rccm.200703-456SO

[2] Silverman EK (2006) Progress in chronic obstructive pulmonary disease genetics. Proc Am Thorac Soc 3: 405–408. 16799082 10.1513/pats.200603-092AW PMC2658703

[3] Cookson WO, Moffatt MF (2011) Genetics of complex airway disease. Proc Am Thorac Soc 8: 149–153. 21543792 10.1513/pats.201101-003MS PMC3131831

[4] Mall MA, Harkema JR, Trojanek JB, Treis D, Livraghi A, et al (2008) Development of chronic bronchitis and emphysema in β-epithelial Na^+^ channel-overexpressing mice. Am J Respir Crit Care Med 177: 730–742. 18079494 10.1164/rccm.200708-1233OC PMC2277210

[5] Knowles MR, Boucher RC (2002) Mucus clearance as a primary innate defense mechanism for mammalian airways. J Clin Invest 109: 571–577. 11877463 10.1172/JCI15217 PMC150901

[6] Mall MA (2008) Role of cilia, mucus, and airway surface liquid in mucociliary dysfunction: lessons from mouse models. J Aerosol Med Pulm Drug Deliv 21: 13–24. 18518828 10.1089/jamp.2007.0659

[7] Goodman RM, Yergin BM, Landa JF, Golivanux MH, Sackner MA (1978) Relationship of smoking history and pulmonary function tests to tracheal mucous velocity in nonsmokers, young smokers, ex-smokers, and patients with chronic bronchitis. Am Rev Respir Dis 117: 205–214. 637405 10.1164/arrd.1978.117.2.205

[8] Hogg JC, Timens W (2009) The pathology of chronic obstructive pulmonary disease. Annu Rev Pathol 4: 435–459. 18954287 10.1146/annurev.pathol.4.110807.092145

[9] Welsh MJ (1983) Cigarette smoke inhibition of ion transport in canine tracheal epithelium. J Clin Invest 71: 1614–1623. 6863537 10.1172/JCI110917 PMC370367

[10] Cantin AM, Hanrahan JW, Bilodeau G, Ellis L, Dupuis A, et al (2006) Cystic fibrosis transmembrane conductance regulator function is suppressed in cigarette smokers. Am J Respir Crit Care Med 173: 1139–1144. 16497995 10.1164/rccm.200508-1330OC

[11] Clunes LA, Davies CM, Coakley RD, Aleksandrov AA, Henderson AG, et al (2012) Cigarette smoke exposure induces CFTR internalization and insolubility, leading to airway surface liquid dehydration. FASEB J 26: 533–545. 21990373 10.1096/fj.11-192377 PMC3290447

[12] Sloane PA, Shastry S, Wilhelm A, Courville C, Tang LP, et al (2012) A pharmacologic approach to acquired cystic fibrosis transmembrane conductance regulator dysfunction in smoking related lung disease. PLoS ONE 7: e39809. 22768130 10.1371/journal.pone.0039809 PMC3387224

[13] Wielputz MO, Eichinger M, Zhou Z, Leotta K, Hirtz S, et al (2011) In vivo monitoring of cystic fibrosis-like lung disease in mice by volumetric computed tomography. Eur Respir J 38: 1060–1070. 21478215 10.1183/09031936.00149810

[14] Zhou L, Dey CR, Wert SE, DuVall MD, Frizzell RA, et al (1994) Correction of lethal intestinal defect in a mouse model of cystic fibrosis by human CFTR. Science 266: 1705–1708. 7527588 10.1126/science.7527588

[15] Mall M, Grubb BR, Harkema JR, O'Neal WK, Boucher RC (2004) Increased airway epithelial Na^+^ absorption produces cystic fibrosis-like lung disease in mice. Nat Med 10: 487–493. 15077107 10.1038/nm1028

[16] Zhou Z, Duerr J, Johannesson B, Schubert SC, Treis D, et al (2011) The ENaC-overexpressing mouse as a model of cystic fibrosis lung disease. J Cyst Fibros 10 Suppl 2S172–S182. 21658636 10.1016/S1569-1993(11)60021-0

[17] Anagnostopoulou P, Dai L, Schatterny J, Hirtz S, Duerr J, et al (2010) Allergic airway inflammation induces a pro-secretory epithelial ion transport phenotype in mice. Eur Respir J 36: 1436–1447. 20413543 10.1183/09031936.00181209

[18] Mall M, Bleich M, Greger R, Schreiber R, Kunzelmann K (1998) The amiloride inhibitable Na^+^ conductance is reduced by CFTR in normal but not in cystic fibrosis airways. J Clin Invest 102: 15–21. 9649552 10.1172/JCI2729 PMC509060

[19] Roth EK, Hirtz S, Duerr J, Wenning D, Eichler I, et al (2011) The K^+^ channel opener 1-EBIO potentiates residual function of mutant CFTR in rectal biopsies from cystic fibrosis patients. PLoS ONE 6: e24445. 21909392 10.1371/journal.pone.0024445 PMC3164200

[20] Harkema JR, Plopper CG, Hyde DM, St George JA (1987) Regional differences in quantities of histochemically detectable mucosubstances in nasal, paranasal, and nasopharyngeal epithelium of the bonnet monkey. J Histochem Cytochem 35: 279–286. 2434556 10.1177/35.3.2434556

[21] Zhou Z, Treis D, Schubert SC, Harm M, Schatterny J, et al (2008) Preventive but not late amiloride therapy reduces morbidity and mortality of lung disease in βENaC-overexpressing mice. Am J Respir Crit Care Med 178: 1245–1256. 18849497 10.1164/rccm.200803-442OC

[22] Scherle W (1970) A simple method for volumetry of organs in quantitative stereology. Mikroskopie 26: 57–60. 5530651

[23] Dunnill MS (1962) Quantitative methods in the study of pulmonary pathology. Thorax 17: 320–328. 25269161 10.1136/thx.17.4.320 PMC1018718

[24] Pfaffl MW (2001) A new mathematical model for relative quantification in real-time RT-PCR. Nucleic Acids Res 29: E45. 11328886 10.1093/nar/29.9.e45 PMC55695

[25] McNicholas CM, Canessa CM (1997) Diversity of channels generated by different combinations of epithelial sodium channel subunits. J Gen Physiol 109: 681–692. 9222895 10.1085/jgp.109.6.681 PMC2217047

[26] Mall MA, Button B, Johannesson B, Zhou Z, Livraghi A, et al (2010) Airway surface liquid volume regulation determines different airway phenotypes in Liddle compared with βENaC-overexpressing mice. J Biol Chem 285: 26945–26955. 20566636 10.1074/jbc.M110.151803 PMC2930694

[27] Rock JR, O'Neal WK, Gabriel SE, Randell SH, Harfe BD, et al (2009) Transmembrane protein 16A (TMEM16A) is a Ca^2+^-regulated Cl^−^ secretory channel in mouse airways. J Biol Chem 284: 14875–14880. 19363029 10.1074/jbc.C109.000869 PMC2685669

[28] Stutts MJ, Canessa CM, Olsen JC, Hamrick M, Cohn JA, et al (1995) CFTR as a cAMP-dependent regulator of sodium channels. Science 269: 847–850. 7543698 10.1126/science.7543698

[29] Hopf A, Schreiber R, Mall M, Greger R, Kunzelmann K (1999) Cystic fibrosis transmembrane conductance regulator inhibits epithelial Na^+^ channels carrying Liddle's syndrome mutations. J Biol Chem 274: 13894–13899. 10318798 10.1074/jbc.274.20.13894

[30] Livraghi-Butrico A, Grubb BR, Kelly EJ, Wilkinson KJ, Yang H, et al (2012) Genetically determined heterogeneity of lung disease in a mouse model of airway mucus obstruction. Physiol Genomics 44: 470–484. 22395316 10.1152/physiolgenomics.00185.2011 PMC3339860

[31] Mall M, Hipper A, Greger R, Kunzelmann K (1996) Wild type but not ΔF508 CFTR inhibits Na^+^ conductance when coexpressed in *Xenopus* oocytes. FEBS Lett 381: 47–52. 8641437 10.1016/0014-5793(96)00079-8

[32] Rubenstein RC, Lockwood SR, Lide E, Bauer R, Suaud L, et al (2011) Regulation of endogenous ENaC functional expression by CFTR and DeltaF508-CFTR in airway epithelial cells. Am J Physiol Lung Cell Mol Physiol 300: L88–L101. 20935229 10.1152/ajplung.00142.2010 PMC3023291

[33] Mall MA (2009) Role of the amiloride-sensitive epithelial Na^+^ channel in the pathogenesis and as a therapeutic target for cystic fibrosis lung disease. Exp Physiol 94: 171–174. 19060118 10.1113/expphysiol.2008.042994

[34] Grubb BR, O'Neal WK, Ostrowski LE, Kreda SM, Button B, et al (2012) Transgenic hCFTR expression fails to correct β-ENaC mouse lung disease. Am J Physiol Lung Cell Mol Physiol 302: L238–L247. 22003093 10.1152/ajplung.00083.2011 PMC3349361

[35] Duerr J, Gruner M, Schubert SC, Haberkorn U, Bujard H, et al (2011) Use of a new-generation reverse tetracycline transactivator system for quantitative control of conditional gene expression in the murine lung. Am J Respir Cell Mol Biol 44: 244–254. 20395635 10.1165/rcmb.2009-0115OC

[36] Collawn JF, Lazrak A, Bebok Z, Matalon S (2012) The CFTR and ENaC Debate – How Important is ENaC in CF Lung Disease? Am J Physiol Lung Cell Mol Physiol 302: L1141–6. 22492740 10.1152/ajplung.00036.2012 PMC3379041

[37] Welsh MJ, Ramsey BW, Accurso F, Cutting GR (2001) Cystic fibrosis. In: Scriver CR, Beaudet AL, Sly WS, and Valle D, editors. The Metabolic & Molecular Bases of Inherited Disease. New York: McGraw-Hill. 5121–5188.

[38] Kunzelmann K, Mall M (2001) Pharmacotherapy of the ion transport defect in cystic fibrosis. Clin Exp Pharmacol Physiol 28: 857–867. 11703384 10.1046/j.1440-1681.2001.03541.x

[39] Sly PD, Brennan S, Gangell C, de Klerk N, Murray C, et al (2009) Lung disease at diagnosis in infants with cystic fibrosis detected by newborn screening. Am J Respir Crit Care Med 180: 146–152. 19372250 10.1164/rccm.200901-0069OC

[40] Knowles MR, Paradiso AM, Boucher RC (1995) In vivo nasal potential difference: techniques and protocols for assessing efficacy of gene transfer in cystic fibrosis. Hum Gene Ther 6: 445–455. 7542031 10.1089/hum.1995.6.4-445

[41] Boucher RC (2004) Relationship of airway epithelial ion transport to chronic bronchitis. Proc Am Thorac Soc 1: 66–70. 16113415 10.1513/pats.2306018

[42] Bodas M, Min T, Mazur S, Vij N (2011) Critical modifier role of membrane-cystic fibrosis transmembrane conductance regulator-dependent ceramide signaling in lung injury and emphysema. J Immunol 186: 602–613. 21135173 10.4049/jimmunol.1002850 PMC3119853

[43] Bodas M, Min T, Vij N (2011) Critical role of CFTR-dependent lipid rafts in cigarette smoke-induced lung epithelial injury. Am J Physiol Lung Cell Mol Physiol 300: L811–L820. 21378025 10.1152/ajplung.00408.2010 PMC3119127

[44] Teichgraber V, Ulrich M, Endlich N, Riethmuller J, Wilker B, et al (2008) Ceramide accumulation mediates inflammation, cell death and infection susceptibility in cystic fibrosis. Nat Med 14: 382–391. 18376404 10.1038/nm1748

[45] Petrache I, Natarajan V, Zhen L, Medler TR, Richter AT, et al (2005) Ceramide upregulation causes pulmonary cell apoptosis and emphysema-like disease in mice. Nat Med 11: 491–498. 15852018 10.1038/nm1238 PMC1352344

[46] Van Goor F, Hadida S, Grootenhuis PD, Burton B, Cao D, et al (2009) Rescue of CF airway epithelial cell function in vitro by a CFTR potentiator, VX-770. Proc Natl Acad Sci USA 106: 18825–18830. 19846789 10.1073/pnas.0904709106 PMC2773991

[47] Accurso FJ, Rowe SM, Clancy JP, Boyle MP, Dunitz JM, et al (2010) Effect of VX-770 in persons with cystic fibrosis and the G551D-CFTR mutation. N Engl J Med 363: 1991–2003. 21083385 10.1056/NEJMoa0909825 PMC3148255

[48] Ramsey BW, Davies J, McElvaney NG, Tullis E, Bell SC, et al (2011) A CFTR potentiator in patients with cystic fibrosis and the G551D mutation. N Engl J Med 365: 1663–1672. 22047557 10.1056/NEJMoa1105185 PMC3230303

[49] Becq F, Mall MA, Sheppard DN, Conese M, Zegarra-Moran O (2011) Pharmacological therapy for cystic fibrosis: from bench to bedside. J Cyst Fibros 10 Suppl 2S129–S145. 21658632 10.1016/S1569-1993(11)60018-0

[50] Peters LL, Robledo RF, Bult CJ, Churchill GA, Paigen BJ, et al (2007) The mouse as a model for human biology: a resource guide for complex trait analysis. Nat Rev Genet 8: 58–69. 17173058 10.1038/nrg2025

