# Supplementary figures and images for: CFTR Regulates Early Pathogenesis of Chronic Obstructive Lung Disease in βENaC-Overexpressing Mice
Source: PLoS One. 2012 Aug 24;7(8):e44059. doi: 10.1371/journal.pone.0044059 (PMC3427321; doi:10.1371/journal.pone.0044059)

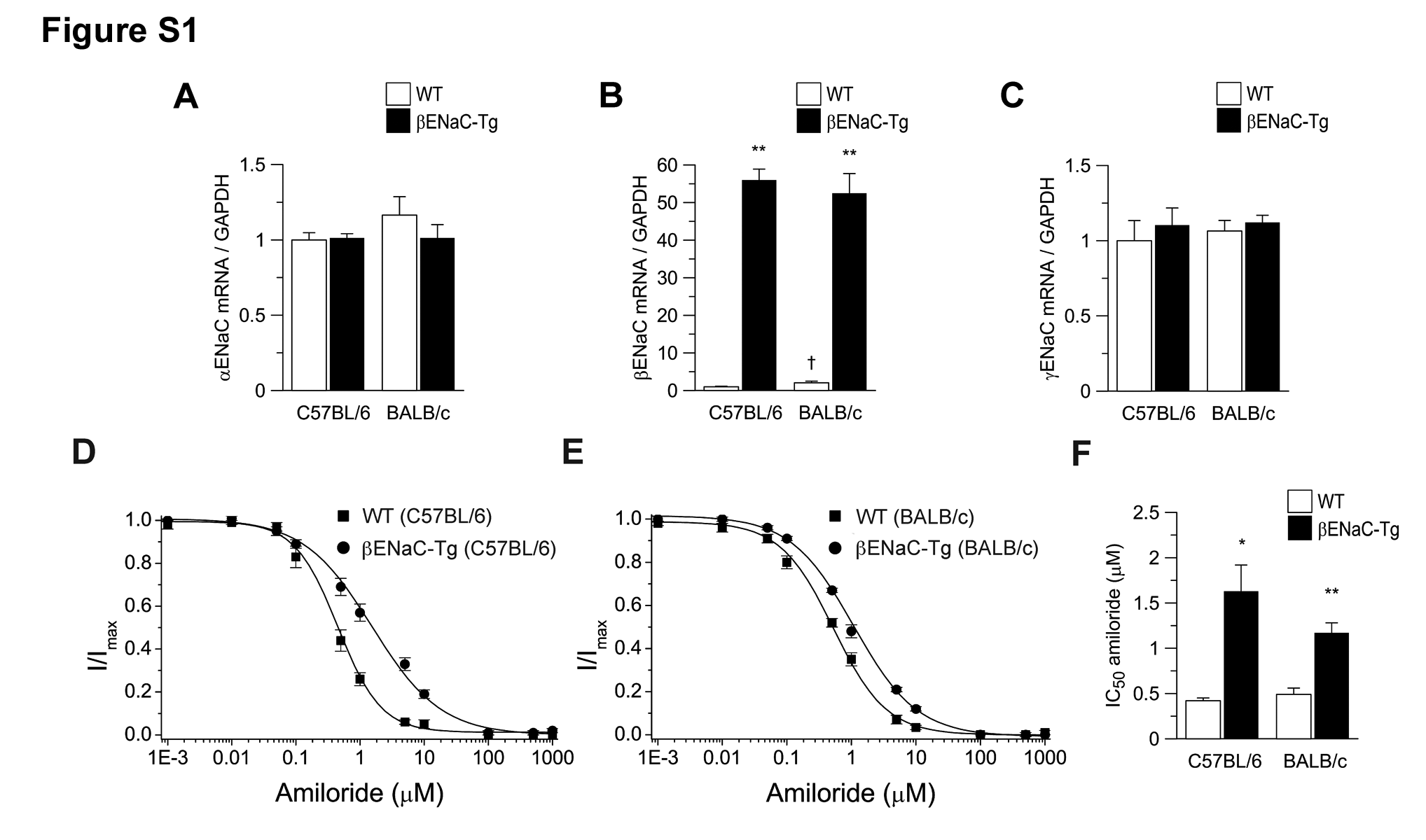

Supplement: Figure S1 — Genetic background has no effect on α, β and γENaC expression and ENaC-mediated Na+ transport in airways of wild-type and βENaC-Tg mice. A–C) Transcript levels of αENaC (A), βENaC (B) and γENaC (C) in freshly excised tracheal tissues from neonatal wild-type (WT) and βENaC-Tg mice on the C57BL/6 and BALB/c background. Data are expressed as fold changes from WT mice on the C57BL/6 background (n = 4–5 samples pooled from 16–20 mice per group). D–F) Amiloride dose-response curves (D,E) and summary of IC50 values (F) obtained from tracheal tissues of neonatal WT and βENaC-Tg mice on the C57BL/6 and BALB/c background (n = 6–9 mice per group). Data are presented as mean ± SEM. *P<0.01 and **P<0.001 compared with WT mice on same strain background, † P<0.05 compared with mice of same genotype on C57BL/6 background. (TIF) [file pone.0044059.s001.tif]

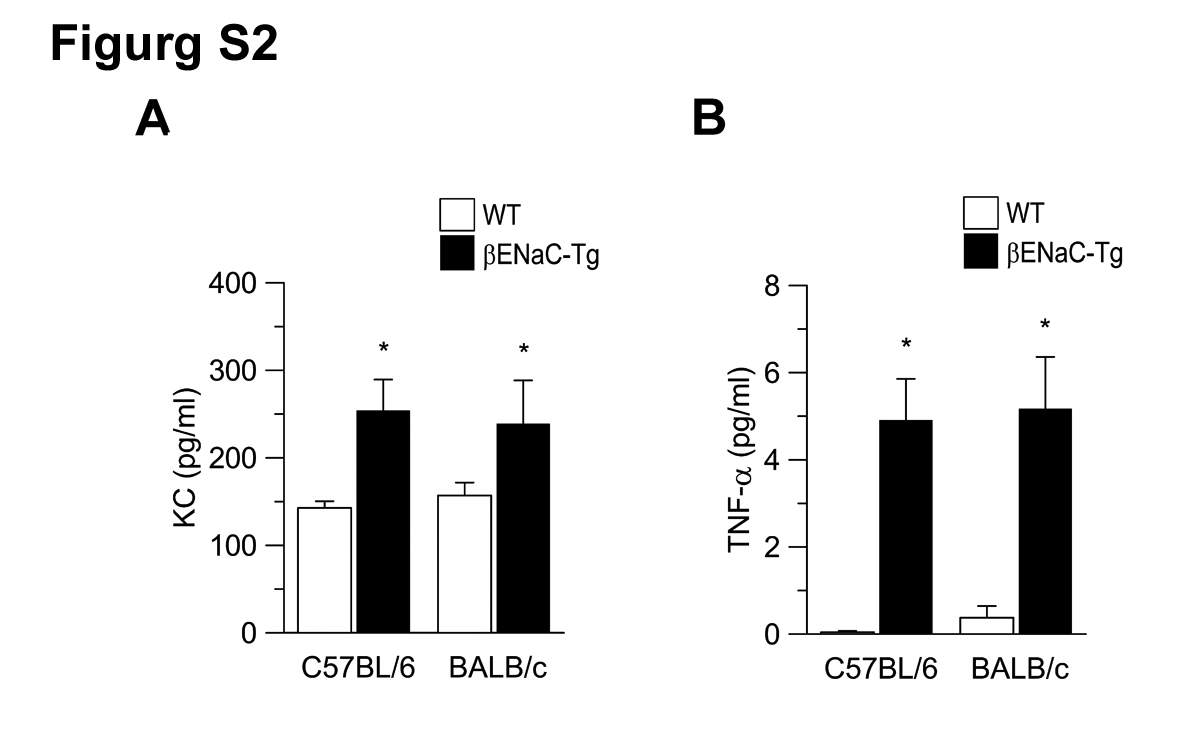

Supplement: Figure S2 — Genetic background has no effect on early airway inflammation in neonatal βENaC-Tg mice. A, B) Levels of KC (A) and TNF-α (B) in lung homogenates from 3-day-old neonatal wild-type (WT) and βENaC-Tg mice on the C57BL/6 and BALB/c background (n = 5–10 mice per group). Data are presented as mean ± SEM. *P<0.05 compared with WT mice on same strain background. (TIF) [file pone.0044059.s002.tif]

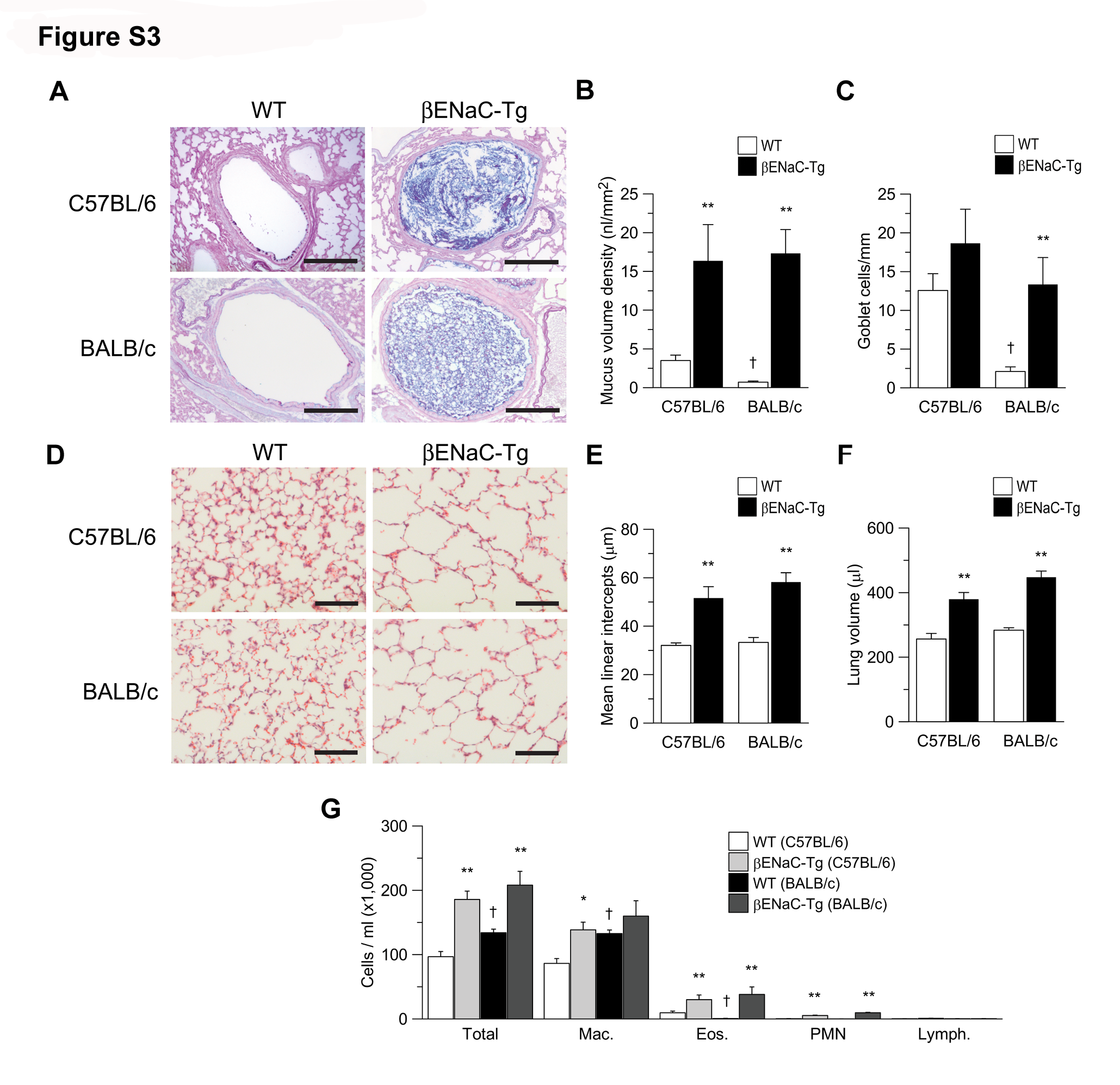

Supplement: Figure S3 — Genetic background has no effect on chronic lung disease in adult βENaC-Tg mice. A) Representative airway morphology of adult (3-week-old) wild-type (WT) and βENaC-Tg mice on the C57BL/6 and BALB/c background. Sections were stained with Alcian blue-periodic acid Schiff (AB-PAS) to determine the presence of airway mucus and goblet cells. Scale bars = 200 µm. B,C) Summary of mucus content, as determined from measuring volume density of AB-PAS-positive material in the airway lumen (B), and goblet cell numbers (C) (n = 9–20 mice per group). D) Representative morphology of distal airspaces. Sections were stained with hematoxylin and eosin (H&E). Scale bars = 100 µm. E–G) Summary of mean linear intercepts (E), lung volume (F), and total and differential cell counts in bronchoalveolar lavage (BAL) fluid (G) from adult WT and βENaC-Tg mice on the C57BL/6 and BALB/c background (n = 5–18 mice per group). Data are presented as mean ± SEM. *P<0.05 and **P<0.001 compared with WT mice on same strain background, † P<0.001 compared with mice of same genotype on C57BL/6 background. (TIF) [file pone.0044059.s003.tif]

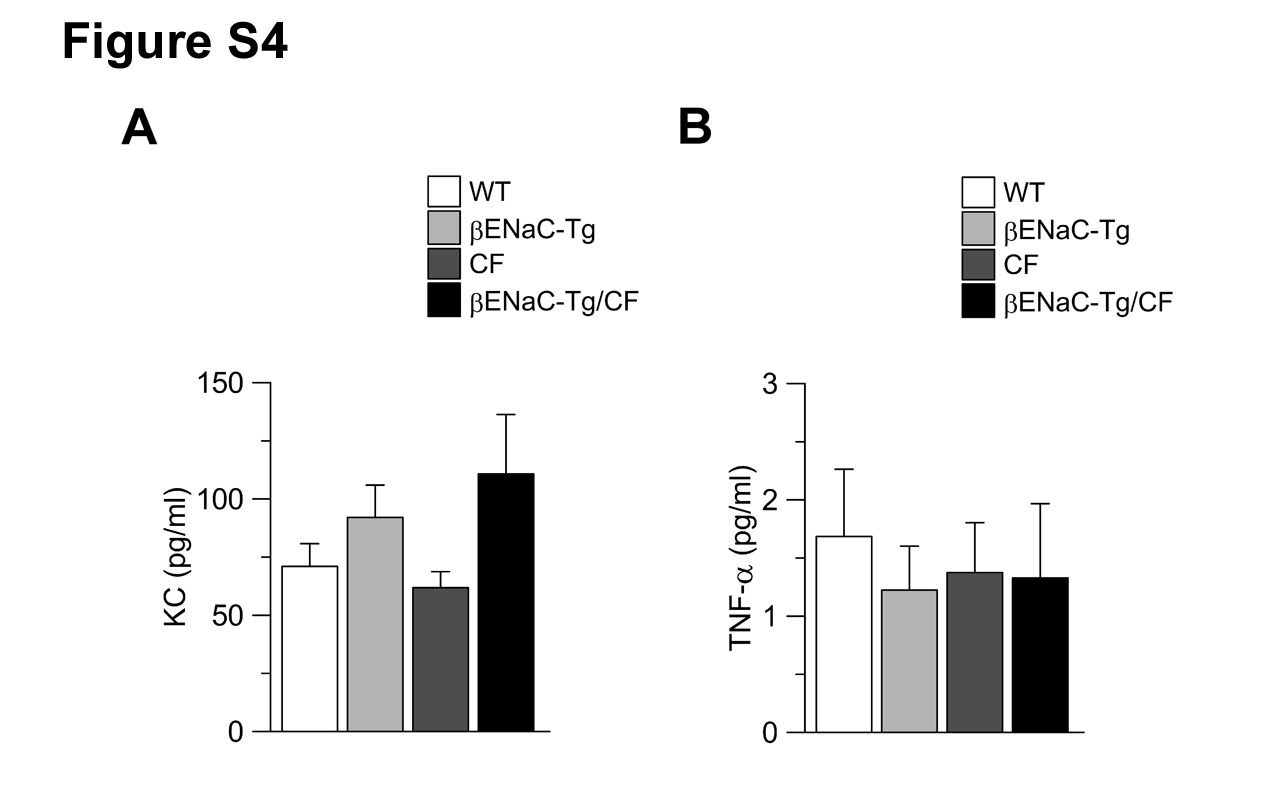

Supplement: Figure S4 — Lack of CFTR does not cause airway inflammation in neonatal mice. A, B) Levels of KC (A) and TNF-α (B) in lung homogenates from newborn (PN 0.5) wild-type (WT), βENaC-Tg, CFTR-deficient (CF) and double mutant βENaC-Tg/CF mice (n = 5–8 mice per group). Data are presented as mean ± SEM. No significant differences were detected between the four experimental groups. (TIF) [file pone.0044059.s004.tif]
